# Third-look contrast-enhanced ultrasonography plus needle biopsy for differential diagnosis of magnetic resonance imaging-only detected breast lesions

**DOI:** 10.1007/s10396-023-01298-8

**Published:** 2023-03-11

**Authors:** Tomohiro Miyake, Kenzo Shimazu

**Affiliations:** Department of Breast and Endocrine Surgery, Osaka University Graduate School of Medicine, 2-2-E10 Yamada-Oka, Suita-Shi, Osaka, 565-0871 Japan

**Keywords:** Breast cancer, MRI-only detected breast lesions, Contrast-enhanced ultrasonography, Second-look ultrasonography, Needle biopsy

## Abstract

Research has shown that in approximately 20–30% of cases, breast lesions that were not detected on mammography (MG) or ultrasonography (US) were incidentally found during preoperative magnetic resonance imaging (MRI) examination for breast cancer. MRI-guided needle biopsy is recommended or considered for such MRI-only detected breast lesions invisible on second-look US, but many facilities in Japan cannot perform this biopsy procedure because it is expensive and time consuming. Thus, a simpler and more accessible diagnostic method is needed. Two studies to date have shown that third-look contrast-enhanced US (CEUS) plus needle biopsy for MRI-only detected breast lesions (i.e., MRI + /MG-/US-) that were not detected on second-look US showed moderate/high sensitivity (57.1 and 90.9%) and high specificity (100.0% in both studies) with no severe complications. In addition, the identification rate was higher for MRI-only lesions with a higher MRI BI-RADS category (i.e., category 4/5) than for those with a lower category (i.e., category 3). Despite the fact that there are limitations in our literature review, CEUS plus needle biopsy is a feasible and convenient diagnostic tool for MRI-only lesions invisible on second-look US and is expected to reduce the frequency of MRI-guided needle biopsy. When third-look CEUS does not reveal MRI-only lesions, a further indication for MRI-guided needle biopsy should be considered according to the BI-RADS category.

## Introduction

Breast magnetic resonance imaging (MRI) examination has high sensitivity and low specificity and is widely performed for preoperative evaluation of breast cancer spread, qualitative diagnosis of breast lesions already identified on other imaging modalities such as mammography (MG) or ultrasonography (US), and breast surveillance for patients with pathogenic variants in breast cancer susceptibility genes. MRI-only detected breast lesions that are not identified on MG or non-enhanced US (i.e., MRI + /MG-/US-) and have a possibility of being malignant may be observed preoperatively; however, the false-positive rate of MRI-only lesions is reportedly high [1–5]. In particular, MRI-only lesions in other segments of the ipsilateral breast or contralateral breast may alter the surgical treatment of breast cancer, and indeed, preoperative MRI examination has been reported to increase the mastectomy rate [6]. Therefore, addition of radiological and pathological diagnosis of MRI-only detected breast lesions is necessary to avoid overtreatment.

Second-look non-enhanced US and US-guided needle biopsy are often performed as initial tests for MRI-only detected breast lesions, and a recent report demonstrated that addition of shear wave elastography improved the specificity of second-look US [7]. However, the detection rate of MRI-only lesions using these techniques varies between facilities [8]. Thus, the possibility of malignancy of MRI-only lesions cannot be ruled out even if second-look US does not reveal them.

Because MRI-guided needle biopsy is a time-consuming and expensive diagnostic procedure, the number of facilities that can provide it is still limited in Japan. We previously reported that the use of a computer-aided detection system may shorten the duration of MRI-guided breast biopsy; however, the results are still preliminary [9]. Real-time virtual sonography using MRI/US fusion reportedly improves the identification rate of MRI-only lesions [10–12], but it requires an additional supine MRI exam and special equipment. Thus, a simpler and more convenient diagnostic method for MRI-only lesions invisible on second-look US is needed.

New diagnostic methods, such as contrast-enhanced US (CEUS) using SonoVue^®^ or Sonazoid^®^, reportedly show favorable sensitivity and specificity for differentiating breast lesions compared with non-enhanced US [13–17]. We herein provide a literature review of the utility of third-look CEUS and CEUS-guided needle biopsy for the diagnosis of MRI-only detected breast lesions invisible on second-look non-enhanced US as an alternative to MRI-guided needle biopsy.

### Prevalence of MRI-only detected breast lesions in preoperative setting

The identification rate of MRI-only detected breast lesions before breast cancer surgery reportedly ranges from 18.8 to 31.2% overall, from 10.7 to 14.0% in the ipsilateral breast, and from 4.9 to 16.0% in the contralateral breast (Table 1) [1–5]. In addition, the frequency of cancer in MRI-only lesions tends to be higher in the ipsilateral breast (12.2–85.7%) than in the contralateral breast (6.8–25.0%). Preoperative MRI reportedly contributes to accurate surgical planning [18, 19]. Further, addition of preoperative MRI to MG and US was reported to increase synchronous cancer detection and contribute to a decrease in metachronous contralateral breast cancer [20, 21]. Based on these findings, bilateral breast MRI has become established as an essential preoperative examination.

**Table 1:** Prevalence of preoperative MRI-only detected breast lesions

Authors	Year	No. of patients	No. of MRI-only lesions	Identification rate of MRI-only lesions in all patients (%)			Location of MRI-only lesions from the index tumor (%)		Malignancy rate of MRI-only lesions (%)	
				Total	Ipsilateral	Contralateral	Ipsilateral	Contralateral	Ipsilateral	Contralateral
Lee [1]	2020	1252	429	31.2	NA	NA	30.5	69.5	12.2	8.7
Brück [2]	2018	50	15	28.0	14.0	16.0	46.7	53.3	85.7	12.5
Cheung [3]	2015	312	85	26.9	NA	NA	74.1	25.9	57.1	22.7
Saha [4]	2015	425	114	18.8	13.9	4.9	78.9	21.1	37.8	25.0
Kim [5]	2014	1038	243	22.0	10.7	12.7	45.7	54.3	18.9	6.8

*MRI* magnetic resonance imaging, *NA* not available

Lesions detected on MRI are categorized according to the Breast Imaging Reporting and Data System (BI-RADS) [22]. Examination by needle biopsy under MRI guidance is recommended for lesions of BI-RADS category 4 (suspicious for malignancy with a > 2% to < 95% probability of malignancy) and those of BI-RADS category 5 (highly suggestive of malignancy with a ≥ 95% probability of malignancy). In contrast, the malignancy rate of BI-RADS category 3 lesions (probably benign) is highly variable among previous studies, although the pooled malignancy rate meets the BI-RADS benchmark (≤ 2%) [23]. Thus, there is no consensus that further imaging and needle biopsy can be omitted for BI-RADS category 3 MRI-only lesions before surgery, especially if the surgical procedure changes according to the pathological diagnosis of them.

### Identification rate of MRI-only detected breast lesions by means of second-look US

Second-look US is a noninvasive and simple diagnostic method for MRI-only detected breast lesions (Table 2) [2, 3, 24–27]. Of all MRI-only lesions, 56.9 to 84.7% can be identified with second-look US, and the frequency of cancer in MRI-only lesions ranges from 21.8 to 56.9%. In detail, the identification rate of MRI-only lesions by means of second-look US was reported to be 88.9% in BI-RADS category 5 MRI-only lesions, 72.7% in BI-RADS category 4 MRI-only lesions, and 75.0% in BI-RADS category 3 MRI-only lesions in a study by Luciani et al. [25], and 67.2% in BI-RADS category 4/5 MRI-only lesions in a study by Candelaria et al. [26]. The former report suggests that MRI-only detected breast lesions of higher BI-RADS categories are detected on second-look US more frequently than those of lower BI-RADS categories. The identification rate of MRI-only lesions finally diagnosed as malignant and benign ranges from 61.4 to 100.0% and from 51.9 to 75.0%, respectively. The widely ranging identification rates of malignant MRI-only lesions among previous reports suggest that further radiological and pathological diagnosis cannot be omitted even if second-look US does not reveal MRI-only lesions, although second-look US can more frequently identify malignant than benign lesions in most of the cases.

**Table 2:** Identification rate of MRI-only detected breast lesions using second-look ultrasonography

Authors	Year	No. of MRI-only lesions examined using SLUS	Malignancy rate of MRI-only lesions (%)	Identification rate of MRI-only lesions using SLUS (%)		
				Total	Malignant lesions	Benign lesions
Brück [2]	2018	9	44.4	77.8	100.0	60.0
Cheung [3]	2015	85	56.9	84.7	100.0	70.5
Laguna [24]	2011	123	30.1	61.8	70.3	58.1
Luciani [25]	2011	55	56.4	76.4	77.4	75.0
Candelaria [26]	2011	131	31.3	67.2	61.4	70.1
Abe [27]	2010	202	21.8	56.9	75.0	51.9

*MRI* magnetic resonance imaging, *SLUS* second-look ultrasonography

### Diagnostic utility of CEUS plus needle biopsy for MRI-only detected breast lesions invisible on second-look US

The diagnostic ability of both non-enhanced US and CEUS using SonoVue^®^ or Sonazoid^®^ for differentiating breast tumors is shown in Table 3 [13–17]. The sensitivity and specificity range from 71.1 to 95.3% and 57.7 to 80.6%, respectively, for non-enhanced US and from 75.8 to 95.3% and 82.1 to 96.8%, respectively, for CEUS. The superiority of CEUS over non-enhanced US in the differential diagnosis of breast tumors has raised clinicians’ expectations regarding the high diagnostic utility of CEUS for MRI-only detected breast lesions invisible on second-look US, and the results of two studies have been reported to date [28, 29].

**Table 3:** Comparison of diagnostic capability between non-enhanced US and CEUS for breast lesions

Authors	Year	No. of patients	No. of lesions	Non-enhanced US			CEUS		
				Accuracy (%)	Sensitivity (%)	Specificity (%)	Accuracy (%)	Sensitivity (%)	Specificity (%)
Pan [13]	2020	51	52	80.7	85.0	78.1	96.1	95.0	96.8
Miyamoto [14]	2014	117	117	65.5	83.8	57.7	87.2	91.4	85.4
Du [15]	2012	61	61	80.3	81.8	78.6	78.7	75.8	82.1
Zhao [16]	2010	71	76	75.0	71.1	80.6	90.8	86.7	96.8
Liu [17]	2008	104	104	83.5	95.3	75.0	91.3	95.3	88.3

*US* ultrasonography, *CEUS* contrast-enhanced ultrasonography

Nykänen et al. [28] investigated 10 BI-RADS category 4/5 MRI-only lesions that were examined with third-look CEUS using SonoVue^®^, and Miyake et al. [29] investigated 42 BI-RADS category 3/4/5 MRI-only lesions that were examined with third-look CEUS using Sonazoid^®^. The latter study included MRI-only breast lesions that were incidentally detected during exams for breast cancer (*n* = 27) and those found during exams for bloody nipple discharge, contralateral breast lesions, and ipsilateral breast lesions in a different segment (*n* = 15). The identification rates of MRI-only lesions by means of CEUS in the two studies (50.0% in the former study and 54.8% in the latter) were almost the same (Table 4). In addition, the detection rates of malignant and benign MRI-only lesions were 57.1% and 33.3%, respectively, in the former report and 100.0% and 40.6%, respectively, in the latter, suggesting that malignant MRI-only lesions can be effectively detected with CEUS. The diagnostic performance of CEUS and CEUS plus needle biopsy for MRI-only lesions in these two studies is shown in Table 5. The diagnostic accuracy of CEUS alone was high in both studies, and the addition of needle biopsy to CEUS improved the accuracy. In both studies, no patients reportedly developed complications associated with the contrast media (SonoVue^®^ or Sonazoid^®^). For MRI-only lesions invisible on third-look CEUS, regular follow-up was performed using MG and MRI in the former study (*n* = 1; follow-up period: 12 months) and was performed using physical examination at 3–6 month intervals, annual MG, and using US in at least 6 month intervals with or without breast MRI in the latter (*n* = 10; median follow-up period: 18.5 months; range: 14 − 31 months), respectively.

**Table 4:** Identification rate of MRI-only detected breast lesions invisible on SLUS, using CEUS

Authors	Year	No. of MRI-only lesions undetectable on SLUS	Malignancy rate of MRI-only lesions (%)	Identification rate of MRI-only lesions using CEUS (%)		
				Total	Malignant lesions	Benign lesions
Miyake [29]	2019	42	26.2	54.8	100.0	40.6
Nykänen [28]	2017	10	70.0	50.0	57.1	33.3

*MRI* magnetic resonance imaging, *SLUS* second-look ultrasonography, *CEUS* contrast-enhanced ultrasonography

**Table 5:** Diagnostic accuracy of CEUS alone and CEUS plus needle biopsy for MRI-only detected breast lesions

Authors	No. of MRI-only lesions	CEUS alone			CEUS plus needle biopsy		
		Accuracy (%)	Sensitivity (%)	Specificity (%)	Accuracy (%)	Sensitivity (%)	Specificity (%)
Miyake [29] Nykänen [28]	42 10	71.4 60.0	100.0 57.1	61.3 66.7	97.6 70.0	90.9 57.1	100.0 100.0

*MRI*: magnetic resonance imaging*, CEUS* contrast-enhanced ultrasonography

The 42 MRI-only detected breast lesions in the study conducted by Miyake et al. [29] comprised 18 BI-RADS category 3 MRI-only lesions, 23 category 4 MRI-only lesions, and one category 5 MRI-only lesion. MRI-only lesions of higher categories seemed to show a higher detection rate by means of CEUS than those of lower categories [22.2% (4 of 18) of category 3 vs. 78.3% (18 of 23) of category 4 vs. 100.0% (1 of 1) of category 5 MRI-only lesions]. All of the four BI-RADS category 3 MRI-only lesions visible on CEUS were diagnosed as benign with needle biopsy; one of them was treated by microdochectomy because of continuous bloody nipple discharge and was ultimately upstaged to ductal carcinoma in situ. On the other hand, the rest of the BI-RADS category 3 MRI-only lesions, which were invisible on CEUS, were diagnosed as benign based on the pathological examination at surgery for the index tumor or regular follow-up. Taking these results into consideration, MRI-guided biopsy could be omitted for asymptomatic BI-RADS category 3 MRI-only lesions invisible on third-look CEUS or those diagnosed as benign based on CEUS-guided needle biopsy.

Notably, half of the 18 BI-RADS category 4 MRI-only lesions visible on CEUS were diagnosed as benign based on CEUS-guided needle biopsy, whereas the remaining BI-RADS category 4 MRI-only lesions [which were not detected on CEUS (*n* = 5)] showed no evidence of malignancy during regular follow-up despite the fact that MRI-guided needle biopsy is recommended for BI-RADS category 4 MRI-only lesions invisible on second-look US. In Nykänen’s series [28], one BI-RADS category 4/5 MRI-only lesion invisible on third-look CEUS remained stable during regular follow-up without MRI-guided biopsy. In recent reports, BI-RADS category 4 MRI lesions were subdivided into three categories depending on the likelihood of malignancy: 4A (low suspicion for malignancy: > 2% to ≤ 10% possibility of malignancy), 4B (moderate suspicion for malignancy: > 10% to ≤ 50% probability of malignancy), and 4C (high suspicion for malignancy: > 50% to < 95% probability of malignancy) [30, 31]. Future studies are needed to clarify whether MRI-guided needle biopsy can be omitted for BI-RADS category 4A and even 4B MRI-only lesions, which have a comparatively lower possibility of malignancy among category 4 lesions, when they are invisible on third-look CEUS.

One BI-RADS category 5 MRI-only lesion was included in the study conducted by Miyake et al. [29] and was proven to be malignant based on CEUS-guided needle biopsy. As mentioned above, the identification rate is higher among category 4/5 MRI-only lesions than category 3 lesions. If category 5 MRI-only detected breast lesions are not visualized on CEUS, MRI-guided needle biopsy should be performed.

Our literature review has limitations: there are only two retrospective studies with small sample sizes that investigated the diagnostic utility of third-look CEUS for MRI-only detected breast lesions; different contrast agents such as SonoVue^®^ or Sonazoid^®^ were used for CEUS between the studies; the regular follow-up method for MRI-only lesions invisible on CEUS varied between the studies; the regular follow-up period was not sufficient in the studies. To validate the above-mentioned strategy for MRI-only detected breast lesions invisible on third-look CEUS, further studies including more patients with a longer follow-up period considering BI-RADS MRI classifications with subcategorization of category 4 are needed.

## Conclusion

Despite the fact that there are limitations in our literature review, CEUS plus needle biopsy demonstrated moderate/high diagnostic sensitivity and high specificity for MRI-only detected breast lesions invisible on second-look US in the two above-mentioned studies, suggesting that this technique can reduce the frequency of MRI-guided needle biopsy. When third-look CEUS does not reveal MRI-only lesions, a further indication for MRI-guided needle biopsy should be considered according to the BI-RADS category.

## Data Availability

Data sharing not applicable to this article as no datasets were generated or analysed during the current study.
