# Correction: Third-look contrast-enhanced ultrasonography plus needle biopsy for differential diagnosis of magnetic resonance imaging-only detected breast lesions

**DOI:** 10.1007/s10396-024-01496-y

**Published:** 2024-08-24

**Authors:** Tomohiro Miyake, Kenzo Shimazu

**Affiliations:** Department of Breast and Endocrine Surgery, Osaka University Graduate School of Medicine, 2-2-E10 Yamada-Oka, Suita-Shi, Osaka, 565-0871 Japan

**Correction: Journal of Medical Ultrasonics** 10.1007/s10396-023-01298-8

The article “Third‑look contrast‑enhanced ultrasonography plus needle biopsy for differential diagnosis of magnetic resonance imaging‑only detected breast lesions”, written by Tomohiro Miyake and Kenzo Shimazu, was originally published electronically on the publisher’s internet portal on 11 March 2023 without open access. With the society’s decision to opt for Open Choice the copyright of the article changed on 15 August 2024 to © The Author(s) 2023 and the article is forthwith distributed under a Creative Commons Attribution 4.0 International License, which permits use, sharing, adaptation, distribution and reproduction in any medium or format, as long as you give appropriate credit to the original author(s) and the source, provide a link to the Creative Commons licence, and indicate if changes were made. The images or other third party material in this article are included in the article’s Creative Commons licence, unless indicated otherwise in a credit line to the material. If material is not included in the article’s Creative Commons licence and your intended use is not permitted by statutory regulation or exceeds the permitted use, you will need to obtain permission directly from the copyright holder. To view a copy of this licence, visit http://creativecommons.org/licenses/by/4.0.

